# Evidence for horizontal transfer of mitochondrial DNA to the plastid genome in a bamboo genus

**DOI:** 10.1038/srep11608

**Published:** 2015-06-23

**Authors:** Peng-Fei Ma, Yu-Xiao Zhang, Zhen-Hua Guo, De-Zhu Li

**Affiliations:** 1 Key Laboratory for Plant Diversity and Biogeography of East Asia, Kunming Institute of Botany, Chinese Academy of Sciences, Kunming, Yunnan 650201, China; 2 Plant Germplasm and Genomics Center, Germplasm Bank of Wild Species, Kunming Institute of Botany, Chinese Academy of Sciences, Kunming, Yunnan 650201, China

## Abstract

In flowering plants, three genomes (nuclear, mitochondrial, and plastid) coexist and intracellular horizontal transfer of DNA is prevalent, especially from the plastid to the mitochondrion genome. However, the plastid genomes are generally conserved in evolution and have long been considered immune to foreign DNA. Recently, the opposite direction of DNA transfer from the mitochondrial to the plastid genome has been reported in two eudicot lineages. Here we sequenced 6 plastid genomes of bamboos, three of which are neotropical woody species and three are herbaceous ones. Several unusual features were found, including the duplication of *trnT-GGU* and loss of one copy of *rps19* due to contraction of inverted repeats (IRs). The most intriguing was the ~2.7 kb insertion in the plastid IR regions in the three herbaceous bamboos. Furthermore, the insertion was documented to be horizontally transferred from the mitochondrial to the plastid genome. Our study provided evidence of the mitochondrial-to-plastid DNA transfer in the monocots, demonstrating again that this rare event does occur in other angiosperm lineages. However, the mechanism underlying the transfer remains obscure, and more studies in other plants may elucidate it in the future.

Horizontal DNA transfer, the transfer of genetic material between donor and recipient organisms by asexual means, has played a major role in the evolution of bacteria^1^,^2^. Recently, the prevalence and importance of this process in the evolution of eukaryotes is being increasingly recognized, especially in plants^3^,^4^,^5^. In addition to the plant-to-plant horizontal DNA transfer, a large amount of these cases reported involved the transfer of DNA within a cell in the same plant species^6^,^7^,^8^.

In flowering plants, three distinct genomes of nuclear, mitochondrial, and plastid coexist within each individual cell, facilitating the intracellular DNA exchange. However, the direction of DNA transfer between the three genomes is highly uneven^9^. For example, the nuclear genome usually harbors lots of nucleotide sequences derived from the mitochondrial and plastid DNA^7^,^8^,^10^. Similarity, the mitochondrial genome can also accumulate large amounts of nuclear DNA and particularly plastid DNA^11^,^12^,^13^, although the size of mitochondrial genome is generally much smaller than the nuclear genome in angiosperms. By contrast, the plastid genome is nearly immune from the promiscuous DNA and the transfer of DNA from the nuclear and mitochondrial to the plastid genome is very scarce^7^,^9^. There are only two examples of dicots reported in the angiosperms: one from the carrot (*Daucus carota*)^14^,^15^ and the other from the common milkweed (*Asclepias syriaca*)^16^. Both of them are the transfer of DNA from mitochondrion to plastid and the transferred segment is ~1.5 kb and ~2.4–4.7 kb in length in the plastid genomes of carrots and milkweeds^14^,^15^,^16^, respectively. Nevertheless, little is known about the exact mechanisms underlying the transfer^9^, although the non-LTR retrotransposon in the carrots^15^ or homologous recombination through double-strand break repair in the milkweeds^16^ was proposed as the possible mechanism in explaining the transfer.

The plastid genomes of angiosperms are highly conserved in evolution^17^,^18^,^19^. The plastid genomes usually have a circular genome organization, with a pair of inverted repeats (IRs) dividing the whole genome into two single-copy regions: large-single copy (LSC) and small-single copy (SSC) regions. The angiosperms plastid genomes range generally from 120 to 160 kb in size, encoding approximately 80 unique protein-coding genes that are mainly responsible for photosynthesis. And the gene order in the plastid genomes is generally conserved across angiosperm species. Furthermore, the rates of DNA sequence substitution are slow in the plastid genomes and about threefold lower than the nuclear DNA^17^,^20^.

The approximately 1430 species of bamboos around the world comprise of the Bambusoideae, a subfamily in the grass family (Poaceae), which can be divided into three main lineages: temperate woody bamboos (tribe Arundinarieae), tropical woody bamboos (tribe Bambuseae), and herbaceous bamboos (tribe Olyreae)^21^,^22^. The tropical woody bamboos can be further divided into neotropical and paleotropical woody bamboos^21^,^22^. Three subtribes (Buergersiochloinae, Parianinae, and Olyrinae) are recognised within Olyreae, consisting of 21 genera and 122 species^21^. There were 30 complete plastid genomes of bamboos sequenced when the manuscript was submitted, and 29 of them were from the woody bamboos^23^,^24^,^25^. All the sequenced bamboos have the same plastid genome organization and are very similar to each other in nearly every aspect of genome features, only with limited DNA sequence divergence^23^,^24^,^25^.

## Results

### Sequencing of six bamboo plastid genomes

We reported here the complete plastid genome sequences of six bamboos through sequencing of total genomic DNA or PCR-amplified plastid DNA using Illumina technology. Among these plastid genomes, three were from the herbaceous bamboo genus *Pariana*, and the other three were the neotropical woody bamboos: *Chusquea* sp., *C. circinata*, and *Otatea glauca*^21^ (Table 1). The *C. circinata* and *O. glauca* were sequenced on PCR-amplified plastid DNA with 2.45 and 1.96 million quality filtered paired-end reads of 90 bp obtained and an estimated sequencing depth of 4638.7× and 3057.7×, respectively. The remaining four bamboos were sequenced on total DNA, and 1.99, 2.12, 3.07 and 3.12 million quality filtered paired-end reads of 90 bp with an estimated sequencing depth of 41.0×, 23.7×, 14.4× and 30.1× were obtained for *Chusquea* sp., *Pariana* sp., *P. radiciflora* and *P. campestris*, respectively. The four junctions between IRs and LSC/SSC in the sequenced plastid genomes were all validated by Sanger resequencing^24^.

The six newly sequenced plastid genomes of bamboos are very similar to those of previously published bamboos^23^,^24^,^25^ (Table 1). The presence or absence of genes in the genome is the same in these bamboos, except that *Chusquea* sp. has two copies of *trnT-GGU* and all three members of *Pariana* encode only one copy of the *rps19* gene. The 72-bp *trnT-GGU* is duplicated in *Chusquea* sp. associated with a 94-bp tandem repeat, but not in other bamboos. The *rps19* gene near the junctions between IRs and LSC regions is always confined to the IR regions in the sequenced bamboo plastid genomes^23^,^24^,^25^, while in *Pariana* it lies entirely within the LSC region due to shift of the IRs/LSC junctions. In terms of genome size, the lineage of neotropical woody bamboos shows a tendency of reduction in the Bambusoideae, and the reduction is especially evident in the IRs size of *O. glauca* (~20.3 kb versus an average ~21.8 kb in other bamboos) (Table 1). By contrast, the IRs size undergoes expansion (~22.5 kb versus an average ~21.8 kb in other bamboos) in *Pariana* with an overall genome size larger than 140 kb. Although the evolution of the IR regions is very conserved^17^, these newly sequenced plastid genomes were variable in the IRs size. Further investigation revealed that the variation could be attributed to the variability in the length of the intergenic spacer between *trnI-CAU* and *trnL-CAA*.

### Insertion of mitochondrion-derived DNA in the *trnI-CAU*-*trnL-CAA* intergenic spacer

**Figure 1:**
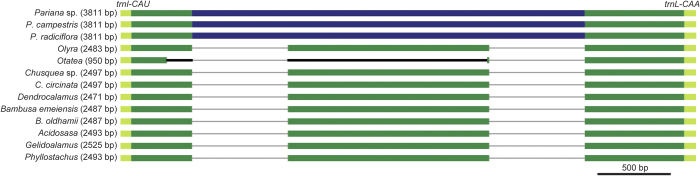
Variability in the *trnI*-*CAU-trnL-CAA* intergenic spacer in the plastid genomes of Bambusoideae with the size indicated in parentheses. The insertion without plastid DNA homology in the plastid genomes of *Pariana* is shown in blue and the other plastid DNA sequences are shown in green. Sequence deletions larger than 25 bp relative to other species are indicated by black lines.

The *trnI-CAU*-*trnL-CAA* intergenic spacer located in the IR regions is very similar in size among the 30 sequenced plastid genomes of bamboos, ranging narrowly from 2471 to 2525 bp. The remnants of the pseudogenized *ycf2* gene that is encoded in most other angiosperms could be identified in this intergenic spacer in grasses^26^. However, sequencing of the six bamboo plastid genomes here revealed extensive variation of this intergenic spacer in length: *Pariana* had the longest (3811 bp) and *O. glauca* the shortest (950 bp) (Fig. 1). By contrast, the size of other intergenic spacers of these bamboos in the IR regions remained fairly constant as in other bamboos. In *O. glauca*, the *trnI-CAU*-*trnL-CAA* intergenic spacer was much reduced to 950 bp because of a large deletion of 1542 bp between nucleotide positions 234 to 1775 when using *C. circinata* as a reference. This spacer also experienced sequence deletion at nearly the same position as in *O. glauca* (positions 421 to 1799 in *C. circinata*) in all three species of *Pariana* (Fig. 1), and the size of the deletion was estimated to be 1379 bp. However, the 1379-bp deletion in *Pariana* was accompanied by a 2706-bp insertion with the total size of the *trnI-CAU*-*trnL-CAA* intergenic spacer expanding to 3811 bp (Fig. 1). In addition, the sequences of insertion differed by only one nucleotide site (A to C substitution) among the three sequenced species of *Pariana*. Although the similarity of sequences surrounding the insertion in the *trnI-CAU*-*trnL-CAA* intergenic spacer was very high (>98%) between *Pariana* and the other sequenced bamboos, the sequence of insertion did not show similarity to any other sequences in the bamboo plastid genomes, suggesting that it perhaps originated from foreign DNA.

To identify the origin of the insertion, the inserted sequence in the plastid genome of *P. campestris* was used as query in a Blastn search against all the sequences deposited in the NCBI nucleotide database. A total of 16 significant hits with >70% similarity to the query over 100 bp were identified and 12 of these with ≥85% similarity were listed in Table 2. These hits were all derived from the mitochondrial DNA sequences of angiosperms, except that a 201-bp segment of the insertion had matching sequences from both mitochondrial and nuclear genomes of *Triticum aestivum* (Table 2). This result strongly suggested that the inserted sequence in plastid genome of *Pariana* originated from the mitochondrial DNA. Furthermore, the hit receiving the highest Blastn score was from the mitochondrial genome of the bamboo *Ferrocalamus rimosivaginus* (Arundinarieae)^27^, covering ~97% sequence of the insertion. The second one was also from another bamboo *Bambusa oldhamii* (Bambuseae), covering ~29% sequence of the insertion. It should here be noted that the *F. rimosivaginus* and *B. oldhamii* are the only two bamboos with mitochondrial genome sequences available in the NCBI nucleotide database to date. The remaining hits all covered less than 20% sequence of the insertion (Table 2). Notably, the first 75 bp sequence of the insertion lacked detectable similarity with any of the plastid, mitochondrial, or nuclear sequences available in the NCBI nucleotide database.

### Validation of the mitochondrion-to-plastid DNA transfer

**Figure 2:**
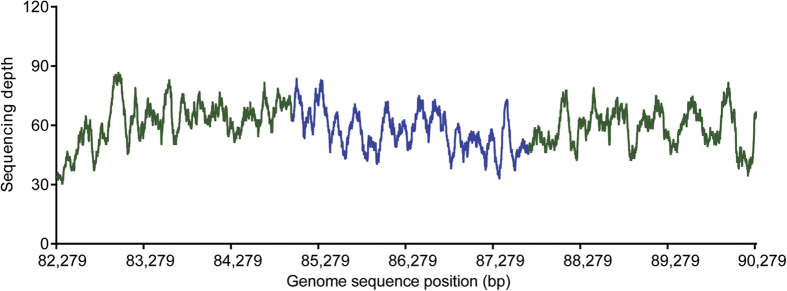
Sequencing depth of the insertion (shown in blue) and its surrounding regions (shown in green) in the plastid genome of *P. campestris*. The position 82,279 is the start site of the inverted repeat regions.

Given that the plastid genomes are highly conserved in bamboos^23^,^24^,^25^, the large insertion being of mitochondrial DNA origin is unexpected. It is needed to confirm that the insertion was not a product of misassembly. As we used total genomic DNA of *Pariana* for Illumina sequencing, the reads were a mixture of DNA sequences from plastid, mitochondrial, and nuclear genomes. However, the plastid genome is usually the smallest one among these three genomes in angiosperms and the assembled plastid DNA sequences can be easily distinguished from mitochondrial and nuclear DNA sequences by sequencing depth. As shown in Fig. 2, the inserted region in *P. campestris* had an average sequencing depth of 56.9×, which was very similar to the surrounding regions with an average sequencing depth of 61.8× and 58.8× before and after the insertion, respectively. Although different sequencing depths of 29.8× and 28.7× were revealed for the inserted regions in *Pariana* sp. and *P. radiciflora*, respectively, the constant sequencing depth in the surrounding regions was also observed in these two bamboos (Supplementary Fig. 1). On the other hand, we mapped the Illumina reads of *P. campestris* to three randomly selected intronless genes from the *F. rimosivaginus* mitochondrial genome^27^, *ccmFN*, *matR* and *rrn26*, and obtained much lower sequencing depth of 5.6×, 7.1× and 11.9×, respectively. The sequencing depth for the three genes was also low in the other two *Pariana* species, ranging from 3.5× to 6.8×. These results indicated that the inserted regions had similar sequencing depth to the surrounding plastid regions, rather than the mitochondrial genome, even in considering that reads originating from the mitochondrial genome may have added a similar sequencing depth as inferred from the three mitochondrial genes to the inserted regions due to sequence similarity. The nuclear genome is much larger than mitochondrial genome and the sequencing depth would be even lower. In all, the analysis of sequencing depth demonstrated that the insertion in the plastid genomes of *Pariana* was unlikely due to misassembly.

We further used PCR amplification and Sanger sequencing to confirm the insertion. PCR amplification using two pairs of primers (Supplemental Table 1) derived from the regions flanking the endpoints of the insertion yielded the predicted junction fragments in all three *Pariana* species but not in the other representative bamboos in Fig. 1. We also performed PCR amplification that was only expected for the bamboos without the insertion. There were no specific amplicons obtained in the three species of *Pariana*, just as predicted, whereas amplifications were successful when using the representative bamboos (Fig. 1) without the insertion as templates. Furthermore, we designed additional 4 pairs of primers to sequence the whole insertion region (Supplemental Table 1). Sanger sequencing of the PCR products from these 6 pairs of primers yielded a total of 3327 bp sequences for each of the three *Pariana* species, which was separately identical to the assembled plastid genomes. Finally, we used PCR to screen *P. parvispica* and *Eremitis parviflora* from the sister genus within the same subtribe Parianinae^21^,^28^ with available DNA materials for the presence of the insertion. However, PCR products were only obtained with 4 pairs of primers (approximately from positions 84,798 to 87,222 in *P. campestris* in Fig. 2 spanning one junction of the insertion) and 2367 and 2430 bp of sequences were generated for *P. parvispica* and *E. parviflora*, respectively. These sequences shared 99% similarity with their counterparts in *P. campestris* (Supplemental Fig. 2).

### Transfer of mitochondrial DNA to the plastid genome in bamboos

**Figure 3:**
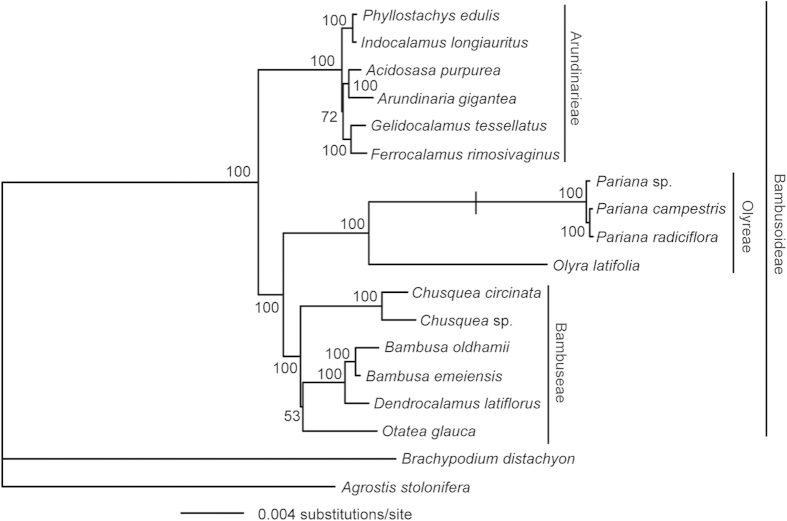
Maximum likelihood phylogeny of the Bambusoideae based on partitioned analysis of the complete plastid genome sequences. The numbers associated with the nodes are bootstrap support values. The hash mark indicates the mitochondrion-to-plastid DNA transfer in the common ancestor of *Pariana*.

To place the mitochondrion-to-plastid DNA transfer in an evolutionary context, we built a phylogenetic tree for the representative species from the three bamboo tribes. Maximum likelihood analyses of the complete plastid genome sequences as a whole and partitioned by the LSC, SSC and IR regions yielded the same topology with nearly identical bootstrap values (Fig. 3 and Supplemental Fig. 3). Each of the three tribes were monophyletic and Olyreae was sister to Bambuseae, supported by 100% bootstrap value (Fig. 3), as in previous studies^22^,^23^. This intracellular DNA transfer was restricted to *Pariana* and its sister genus *Eremitis*, indicating that it likely occurred in their common ancestor.

**Figure 4:**
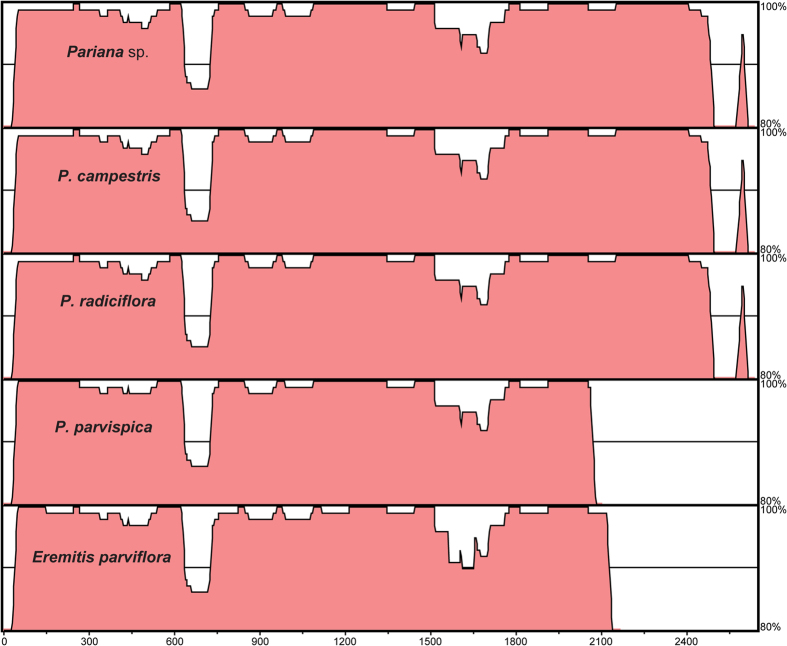
The mVISTA similarity plot of the insertion sequences in the plastid genomes of *Pariana* and *Eremitis* compared with the mitochondrial DNA sequence of *F. rimosivaginus*. The blank areas downstream from ~2.1 kb in *E. parviflora* and *P. parvispica* indicate undetermined sequence of insertion in these bamboos.

The mVISTA^29^ alignment of the mitochondrial DNA sequence of *F. rimosivaginus* and the inserted sequences of 5 Parianinae species revealed a high global level of conservation with sequence identities only falling below 90% between positions approximately 600 and 750 (Fig. 4). Taken *P. campestris* as example, the inserted segment and the *F. rimosivaginus* mitochondrial genome differed by only 25 substitutions (transitions : transversions = 13 : 12) and 9 indels with a total length of 55 bp in an alignment of 2666 bp. Nevertheless, the sequences within 5000 bp upstream and downstream of the matching sequence in the *F. rimosivaginus* mitochondrial genome did not show any similarity to the plastid DNA sequences. The matching sequence is located in the intergenic region between the genes *rps7* and *atp6* in the mitochondrial genome of *F. rimosivaginus*^27^.

To investigate the possible underlying mechanism of integration of mitochondrial DNA into the plastid genome, we performed sequence analysis of the insertion. Similar to the IR regions, the insertion had a GC content that was higher than the LSC/SSC regions of the plastid genome (44.7% compared to 36.2% in *Pariana*). Using ORF (open reading frame) finder in the NCBI (http://www.ncbi.nlm.nih.gov/gorf/gorf.html), we identified a total of 4 ORFs larger than 200 bp with the largest one encoding 116 amino acids. However, these ORFs had no significant similarity to any genes or proteins in the NCBI database. Finally, the insertion was found to be totally devoid of repeated sequences, as well as the 200-bp surrounding sequences in the plastid genome.

## Discussion

The evolution of the reported plastid genomes is fairly conserved among the bamboos^23^,^24^,^25^. In our study, sequencing of six plastid genomes from neotropical woody bamboos and herbaceous bamboo subtribe Parianinae revealed identical genome organization and conserved evolution in bamboos again, but also limited variations. Firstly, the number of encoding genes is different in *Chusquea* sp. and *Pariana*. In *Chusquea* sp., the plastid genome contains two copies of *trnT-GGU* due to a 94-bp tandem repeat. This phenomenon has been repeatedly observed in plant plastid genomes^30^,^31^,^32^, suggesting that repeated sequences may play an important role in generating new genetic material in their evolution. In *Pariana*, the *rps19* gene that is originally located in the IR regions in other bamboos was found to be relocated in the LSC region and thus lose one copy. The junctions between the IR and SSC regions in the plastid genomes are highly variable in grasses with the termini of two genes *ndhF* and *ndhH* repeatedly migrating into and out of the adjacent IR regions^33^. Here we provide evidence that the junctions between the IR and LSC regions can also shift greatly to a degree with the whole *rps19* gene migrating into the LSC region. Secondly and more unexpectedly, there is a large deletion in the IR regions accompanied by insertion derived from the mitochondrial DNA sequence in *Pariana*. And more importantly the insertion was confirmed by analysis of sequencing depth as well as PCR amplification and Sanger sequencing. It is noteworthy that another independent study of plastid genomes of 17 bamboo species including *P. radiciflora* and *Eremitis* sp. also revealed this insertion of horizontally transferred mitochondrial DNA^34^.

In the GenBank, there are presently 446 complete plastid genomes sequenced from angiosperms (last accessed November 11^th^, 2014). However, only plastid genomes from two eudicot lineages have been demonstrated to harbor the horizontal transferred DNA from the mitochondrion^14^,^15^,^16^, even though other published genomes may also contain undiscovered mitochondrion derived DNA sequences^9^. The study of Wysocki *et al.*^34^ and ours focusing on bamboos represent the first example of mitochondrion-to-plastid DNA transfer in monocots (the third in angiosperms), expanding the phylogenetic representation of angiosperms for this phenomenon and confirming again that this direction of intracellular DNA transfer, though very rarely, does occur.

The insertion of mitochondrial DNA origin is located in the *rps2-rpoC2* intergenic spacer in the plastid LSC region in the milkweeds^16^ while in the *rps12-trnV-GAC* intergenic spacer in the plastid IR regions in the carrots^14^,^15^. In the bamboos, it is also located in the intergenic spacer of *trnI-CAU*-*trnL-CAA* in the plastid IR regions. The integration of horizontal transferred DNA into the intergenic regions rather than the coding regions is expected, as the insertion would disrupt gene functions. The insertion occurred in the IR regions in the carrots and bamboos while in the LSC regions in the milkweeds. The high GC content of the transferred DNA (44.7% in *Pariana* and 44.0% in carrots versus 40.3% in milkweeds), which is more similar to the IR regions than to the single-copy regions in the plastid genome^19^, may give an explanation. In addition, the insertion is likely to be accompanied by deletion of plastid sequence at the same region as observed in the carrots (339 bp)^14^,^15^ and here (1379 bp). However, we could not exclude the possibility that the deletion and insertion took place independently in *Pariana* as in the plastid genome of *O. glauca* a deletion of 1542 bp also occurred at almost the same region while without the insertion (Fig. 1). The size of the insertion is ~1.5 kb in the carrots^14^,^15^ and varies between ~2.4–4.7 kb in the milkweeds^16^. The insertion has an identical size of 2.7 kb in the three sequenced bamboos of *Pariana* but we also revealed potential variations in another *Pariana* species and species from the sister genus *Eremitis* as in Wysocki *et al.*^34^. Given that the insertion is likely to be originated from a single event in the common ancestor^14^,^15^,^16^, these results suggest that the transferred sequence may be evolving rapidly. The insertions have been speculated to be transferred as a contiguous sequence from the mitochondria to the plastid genome in the two previously reported examples^14^,^15^,^16^, although they are fragmented into two or three pieces in the corresponding mitochondrial genomes. Our example in which the insertion derived from an intact segment in the mitochondrial genome corroborates this speculation.

While as organelle genomes coexisting in the cell, the ability to incorporating foreign DNA is definitely different between the plastid and mitochondrial genomes in angiosperms^7^,^8^,^9^. Presently, the underlying mechanism is unclear about the integration of foreign DNA into the plastid genome^9^. In the case of *Pariana*, a distinguished genus within Olyreae characterized by showy spike-like inflorescences implying possible insect pollination^21^,^35^, little is known about its genetics and genomics. Although the retrotransposition or homologous recombination has been proposed in previous studies^15^,^16^, we found no evidence in supporting either of these hypotheses in the bamboos. The plastid-to-mitochondrion transferred DNA, such as the multiple tRNA genes of plastid origin^36^, can play an important role in the mitochondrial genome. The role, if any, of the mitochondrion-to-plastid transferred DNA in the evolution of plastid genome otherwise needs more studies to demonstrate in the future.

## Methods section

### Sequencing and assembly of the bamboo plastid genomes

Fresh leaf material was collected from the six bamboos sampled in this study. Among them, *P. campestris* and *P. radiciflora* were collected in the field in French Guiana, *Pariana* sp. was cultivated and kindly provided by Prof. Nian-He Xia (South China Botanical Garden), and the other three bamboos were all cultivated at the Kunming Botanical Garden of the Kunming Institute of Botany. The voucher numbers for the sampled bamboos were DZL14021 for *P. campestris*, DZL14016 for *P. radiciflora*, ZMY026 for *Pariana* sp., ZXZ12008 for *Chusquea* sp., KMBG1217 for *C. circinata*, and KMBG1223 for *O. glauca*.

Total genomic DNA was extracted using the CTAB method^37^ from 0.2–0.5 g fresh material. Total genomic DNA of four bamboos (three *Pariana* species and *Chusquea* sp.) was used for Illumina sequencing. For *C. circinata* and *O. glauca*, the plastid genome was amplified in overlapping fragments using long-range PCR primers from Yang *et al.*^38^, and PCR fragments were pooled together in roughly equal mass as previously described for subsequent sequencing^25^. The DNA samples were sheared to approximately 500 bp at BGI-Shenzhen, followed by library preparation. Paired-end sequencing of 90 bp was conducted on Illumina HiSeq 2000 at BGI-Shenzhen following the manufacturer’s protocol.

Illumina raw reads were first quality trimmed at BGI-Shenzhen. In which reads with adaptor contamination and more than 50% base pairs with a Phred quality score lower than or equal to 5, and having more than 1% ambiguous Ns were all discarded. Subsequently, a combination of de novo assembly using SOAPdenovo v.1.05^39^ and reference-assisted mapping was used to assemble the plastid genomes. The plastid genome of the closely related species *B. emeiensis*^24^ and *Olyra latifolia*^23^ was used as reference for assembly of neotropical woody bamboos and herbaceous bamboos, respectively. SOAPdenovo assemblies were generated with k-mer values of 31, 47, or 81 depended on different data sets. We discarded scaffolds and contigs <1000 bp, and the rest were mapped to the reference genome using Blastn search in NCBI in the default settings. These mapped scaffolds and contigs that had >90% sequences similarity to the reference genome were collected for subsequent genome finishing. The small gaps and/or ambiguous nucleotides in the assembly were resolved by read mapping (see next section). However, the regions representing ~66–83 kb and ~22–33 kb in the reference genome of *B. emeiensis*^24^ could not be assembled for *C. circinata* and *O. glauca*, respectively, due to failure of long-range PCR amplification. Alternatively, we sequenced these regions using PCR primers in previous studies^24^,^25^ or designing new ones if necessary (Supplemental Table S2) by Sanger sequencing. The four junctions between the single-copy and IR regions of the plastid genome were ascertained by Sanger sequencing as previously described^24^. The gene content of the assembled plastid genomes was identified by DOGMA^40^ and manual adjustment was applied if necessary. The annotated plastid genome sequences were deposited in GenBank under accession numbers KP319241-KP319246.

### Analysis of sequencing depth

We mapped the filtered Illumina reads using BWA v.0.7.7^41^ in the default settings to the assembled plastid genomes with one copy of the IRs removed. We only considered these mapped reads that are in properly paired with SAMtools v.0.1.7^42^ and used them to calculate the sequencing depth. For the insertion and surrounding regions, we determined the sequencing depth by averaging the depth at each base extracted using SAMtools. To determine the sequencing depth for the potential assembled mitochondrial DNA sequences, we also mapped the reads to the three mitochondrial genes (*ccmFN*, *matR*, and *rrn26*) of *F. rimosivaginus*^27^ and calculated the depth in the same way.

### Validation of the insertion by PCR amplification

The presence or absence of the insertion in the plastid genome was surveyed by PCR in the three *Pariana* species and in representatives of the major bamboo lineages^21^,^22^ (Fig. 1). Another *Pariana* species (*P. parvispica*) and *E. parviflora* that is sister to *Pariana*^21^,^28^ were also screened. We designed two primer pairs in sequences which are flanking the insertion endpoints and thus diagnostic for the presence of the insertion, and also performed PCR amplification with two primer pairs specific to the plastid DNA sequence without the insertion^25^ (Supplemental Table S1). We confirmed both the endpoints of the insertion in the plastid genomes of *Pariana* sp., *P. campestris*, and *P. radiciflora*. In addition, we also designed 4 PCR primer pairs (Supplemental Table S1) based on the assembled inserted sequence to obtain the whole region.

### Sequence analysis

To determine the origin of the insertion, we conducted Blastn search against the NCBI nucleotide database online. Multiple sequences of the *trnI-CAU*-*trnL-CAA* intergenic spacer were aligned in MEGA5^43^, and manual adjustments were made when necessary. The repetitive sequences were identified by the use of Blastn searches with a minimum identity of 90%.

### Phylogenetic analyses

The complete plastid genome sequences from two species of subfamily Pooideae as outgroups and 16 bamboos (Fig. 3) were aligned in MAFFT v.7.215^44^ with default parameters. A few poorly aligned regions were manually adjusted in MEGA5^43^. We performed unpartitioned and partitioned maximum likelihood analyses of the matrix using RAxML v.8.0.20^45^. In partitioned analysis, the dataset was divided into three partitions corresponding to the LSC, SSC and IR regions of the plastid genome. The maximum likelihood tree was constructed with the combined rapid bootstrap (500 replicates) and search for the best tree in a single run. The GTR + G model was used as suggested in the RAxML manual.

## Additional Information

**How to cite this article**: Ma, P.-F. *et al.* Evidence for horizontal transfer of mitochondrial DNA to the plastid genome in a bamboo genus. *Sci. Rep.* **5**, 11608; doi: 10.1038/srep11608 (2015).

## Supplementary Material

Supplementary Information

## Figures and Tables

**Table 1:** Comparison of plastid genomic characteristics across Bambusoideae. Taxa with plastid genomes sequenced in this study are indicated in bold

**Taxon**	**Size**	**LSC**	**SSC**	**IR**	**Protein Genes (unique)**	**tRNA (unique)**	**rRNA (unique)**	**GC%**
Olyreae								
* Olyra latifolia*	136,785	80,729	12,944	21,556	82(76)	39(31)	8(4)	38.9
* **Pariana*** **sp.**	140,162	82,327	12,743	22,546	83(77)	39(31)	8(4)	38.9
* **P. campestris***	140,120	82,278	12,750	22,546	83(77)	39(31)	8(4)	38.9
* **P. radiciflora***	140,126	82,285	12,749	22,546	83(77)	39(31)	8(4)	38.9
Bambuseae								
Neotropical								
* **Chusquea*** **sp.**	138,257	81,791	12,894	21,786	84(77)	40(32)	8(4)	38.8
* **C. circinata***	137,951	81,431	12,912	21,804	84(77)	39(31)	8(4)	38.8
* **Otatea glauca***	136,377	82,841	12,870	20,333	84(77)	39(31)	8(4)	38.8
Paleotropical								
* Bambusa emeiensis*	139,493	82,988	12,901	21,802	84(77)	39(31)	8(4)	38.9
* B. oldhamii*	139,350	82,889	12,881	21,790	84(77)	39(31)	8(4)	38.9
* Dendrocalamus latiflorus*	139,394	83,030	12,854	21,755	84(77)	39(31)	8(4)	38.9
Arundinarieae								
* Acidosasa purpurea*	139,697	83,273	12,834	21,795	84(77)	39(31)	8(4)	38.9
* Gelidocalamus tessellatus*	139,712	83,220	12,808	21,842	84(77)	39(31)	8(4)	38.9
* Phyllostachys edulis*	139,679	83,213	12,870	21,798	84(77)	39(31)	8(4)	38.9

**Table 2:** Identified DNA sequences homologues to the insertion in the plastid genome of *Pariana* in the Blastn search. All the sequences originate from the angiosperms mitochondrial genomes, except that one from the nuclear genome of *Triticum aestivum*.

**Taxon**	**GenBank accession number**	**Aligned length (bp)**	**Beginning position**	**Ending position**	**Similarity**
*Ferrocalamus rimosivaginus*	JQ235169	2652	76	2706	98%
*Bambusa oldhamii*	EU365401	800	76	874	99%
*Lolium perenne*	JX999996	513	2191	2696	94%
*Amborella trichopoda*	KF754803	246	914	1146	89%
*Hevea brasiliensis*	AP014526	213	2217	2429	91%
*Aegilops speltoides*	AP013107	201	76	276	99%
*Triticum timopheevii*	AP013106	201	76	276	99%
*Triticum aestivum*	AP008982	201	76	276	99%
*Triticum aestivum* (nuclear)	HG670306	201	76	276	98%
*Boea hygrometrica*	JN107812	186	968	1146	85%
*Carica papaya*	EU431224	183	970	1146	88%
*Spirodela polyrhiza*	JQ804980	165	914	1071	91%
