# Supplementary material for: Evidence for horizontal transfer of mitochondrial DNA to the plastid genome in a bamboo genus
Source: Sci Rep. 2015 Jun 23;5:11608. doi: 10.1038/srep11608 (PMC4477325; doi:10.1038/srep11608)
Supplement: Supplementary Information [file srep11608-s1.pdf]

Supplementary Information

Evidence for horizontal transfer of mitochondrial DNA to the plastid genome in a bamboo genus

Peng-Fei Ma[1,2], Yu-Xiao Zhang[1,2], Zhen-Hua Guo[2] & De-Zhu Li[1,2]

[1]Key Laboratory for Plant Diversity and Biogeography of East Asia, Kunming Institute of Botany, Chinese Academy of Sciences, Kunming, Yunnan 650201, China

[2]Plant Germplasm and Genomics Center, Germplasm Bank of Wild Species, Kunming Institute of Botany, Chinese Academy of Sciences, Kunming, Yunnan 650201, China

Correspondence and requests for materials should be addressed to D.Z.L. (dzl@mail.kib.ac.cn)

Table S1. PCR primers used in this study for validation of the insertion in *Pariana*.

Primer	Sequence (5'>3')	Note	Reference
1	F CCTAATTCTCGCTATTTGG	Junction1 of insertion	Present study
	R CTAGGGAGGAAGTAGGAAA		
2	F ATCTGGATGACCGAGAAAT	Junction2 of insertion	Present study
	R GATAATAAAGAACCCAAAGC		
3	F GGTCTCAACTCCCACAAGC	Insertion	Present study
	R CAGAAACCCAGGAAACCAC		
4	F GAATCGTAACCATCCTCCAC	Insertion	Present study
	R CAAGAAGACCAATGAGCACC		
5	F GTAGAAGTGCCAGGATGCG	Insertion	Present study
	R GCTTGTGGGAGTTGAGACC		
6	F CGAAAGCGATGGATGTATGTG	Insertion	Present study
	R TGGCTCAGTCGTGGTGTTATT		
7	F GCCCTGAAAGGAGAAGAAAG	Junction1 without insertion	Present study
	R GAAAACAGAAACCGAGGAAAC		
8	F AGGGATCATCGTACATCGT	Junction2 without insertion	Ref. 25 in paper
	R ATCATTCTGAGCGAGTCCA		

Table S2. PCR primers used in this study for sequencing the plastid genome of *Chusquea circinata* and *Otatea glauca*.

Primer	Sequence (5'>3')	Taxon	Reference
1	F ATGACCAACATAAGGGAAAG	*Chusquea circinata*	Ref. 25 in paper
	R ATACACCCAAAACCACACC		
2	F TCTAATTCGTCCACGCCTAC	*Chusquea circinata*	Ref. 25 in paper
	R ACCCGAGAGAAGCAAAAGTC		
3	F TGTAATGTAGGTTTAGCGGTTC	*Chusquea circinata*	Ref. 25 in paper
	R TGCTGTATCCATGCCTGTTC		
4	F GGGAAAGGTGTTAGAATCAG	*Chusquea circinata*	Ref. 25 in paper
	R GGGAAAGGTGTTAGAATCAG		
5	F CTCTTGATTCGGAATTAGAGA	*Chusquea circinata*	Ref. 25 in paper
	R ATGTAGCGCAGCTTGGTAG		
6	F CCTCGCTACTGCCCATAAA	*Chusquea circinata*	Ref. 25 in paper
	R TGCTTGCGACAATAACGAC		
7	F CTAGACCCAGAAAGAATAGAC	*Chusquea circinata*	Ref. 25 in paper
	R GTAGAGCCAAAGAATGTGA		
8	F TGGCTAAGTAGCTGACCCT	*Chusquea circinata*	Ref. 25 in paper
	R GGTGGCTAATCTCAGGAAT		
9	F CTTGCCCGATAATAAGCAC	*Chusquea circinata*	Ref. 25 in paper
	R TTCCTCCTGAAATACTCCAAC		
10	F GGGATCAGGGATACTTTCAG	*Chusquea circinata*	Ref. 25 in paper
	R AGGGTTTCAATAAACTACCTAC		
11	F ACTCGCGTAGGTAGTTTAT	*Chusquea circinata*	Ref. 25 in paper
	R CTCAAGTTGGCTCATCTGT		
12	F CCGCCCAACCGTTACAGAG	*Chusquea circinata*	Ref. 25 in paper
	R AGAACCGCTAAACCTACATTAC		
13	F TTCATTTCCATTCCCACAAG	*Chusquea circinata*	Ref. 25 in paper
	R CCAATCCGAAGCATAAACA		
14	F GCTTCCGTAACTAAACCTTC	*Chusquea circinata*	Ref. 25 in paper
	R TGATTCAACCTCAGACCCT		
15	F AACATCACCAATACGAGCAT	*Chusquea circinata*	Ref. 25 in paper
	R GTGGGAACGACAGAACCTA		
16	F CGTTCCCACTACTTCTCCT	*Chusquea circinata*	Ref. 25 in paper
	R AAATAAATCCACTCGGTTTC		
17	F GTGTGGGCGTTAGAGCATTG	*Otatea glauca*	Ref. 25 in paper
	R CCTCCCCTACAGTATCGTCAC		
18	F GATTACTCGGGGCGTTCTGTC	*Otatea glauca*	Ref. 25 in paper
	R ATCGGAGCCACAAGGGACTG		
19	F GCTTTGGATGAATCGGTCT	*Otatea glauca*	Ref. 25 in paper
	R AAATGAACTCCCGCTTCTATC		
20	F CCATAGGGATTGATGAAGA	*Otatea glauca*	Ref. 25 in paper
	R ACAAATACGCCCATAGTGAC		
21	F GAGTTGTTTGGGTCCTGGAG	*Otatea glauca*	Ref. 25 in paper

	R	GGCTTGTCGTATCCGTCTG		
22	F	ACCACAGCATTTCTCCCTT	*Otatea glauca*	Ref. 25 in paper
	R	ATCCTGACATTAGAAGTAGCG		
23	F	GGCAAACTATCCAAATCAA	*Otatea glauca*	Ref. 25 in paper
	R	ACAGCAGTATCTACAACCC		
24	F	CGAGGGACTTTCTTTGACAG	*Otatea glauca*	Ref. 25 in paper
	R	GAACCCATTTCTTCGCTTT		
25	F	TGGAATTTTGTCTATCCTTC	*Otatea glauca*	Ref. 25 in paper
	R	TAATATCTTATCCGCCTCT		
26	F	AGGTGGATTGGTCCGAGTG	*Otatea glauca*	Ref. 25 in paper
	R	GGTGAATTATTGCCGATGTC		
27	F	AATTTGAAACAAACCCTCC	*Otatea glauca*	Ref. 25 in paper
	R	TAAAGGTTCTTGTCTTGTGG		
28	F	TATTTCAAGCTATTTCGGCTCT	*Otatea glauca*	Ref. 25 in paper
	R	GTCGGGATGGTTTGTGGAT		
29	F	ATCGGGTGTAGAAGTAGGC	*Otatea glauca*	Ref. 25 in paper
	R	CCAAGCGGATTATCTGGAAC		
30	F	AGGGATCATCGTACATCGT	*Otatea glauca*	Ref. 25 in paper
	R	ATCATTCTGAGCGAGTCCA		
31	F	AGTGGGTCGCTTCTTATGG	*Otatea glauca*	Ref. 25 in paper
	R	TCCTACTGCAAGGGTGGTC		
32	F	ATTGGGTCGTTGCGATTAC	*Otatea glauca*	Ref. 25 in paper
	R	GATGCTTTCGGCTACTGGA		
33	F	GTAGCGGCGAGCGAAATGG	*Otatea glauca*	Ref. 25 in paper
	R	TCACCCTCCGTGGACGAACC		
34	F	CGAAGCGGAAGCGAGAATG	*Otatea glauca*	Ref. 25 in paper
	R	CAGCGTTTACCGTAGGCAC		
35	F	TCTCATGCCTTTCCTCGTTC	*Otatea glauca*	Ref. 25 in paper
	R	GGATCTTCCACCCGTTTAG		
36	F	TCAATTCGGACTGGCTCTT	*Otatea glauca*	Present study
	R	TGGCGCTGCATATTAGAAC		
37	F	CCAAGACGCAGAACAACAG	*Otatea glauca*	Present study
	R	TGGGACTCCGACCATAACA		
38	F	GCAGATCCAGTATAGCGTCTT	*Otatea glauca*	Present study
	R	ATTTCTCCCTTCGTCCATT		
39	F	CGGATGAATTAGTCGTTGT	*Otatea glauca*	Present study
	R	ATGGTGAGTTCCTCGTGTC		
40	F	GACGAAGCGGAAGCGAGAA	*Otatea glauca*	Present study
	R	AAGCCAATCCCAGGGAACA		

Supplementary Figure Legends

Figure S1 | Sequencing depth of the insertion (shown in blue) and its surrounding regions (shown in green) in the plastid genomes of *Pariana* sp. (A) and *P. radiciflora* (B).

Figure S2 | The mVISTA similarity plot of the sequenced insertion and its surrounding sequences in the plastid genomes of *Pariana parvispica* and *Eremitis parviflora* compared with the corresponding sequence of *P. campestris*.

Figure S3 | Maximum likelihood phylogeny of the Bambusoideae based on unpartitioned analysis of the complete plastid genome sequences. The numbers associated with the nodes are bootstrap support values.

Figure S1

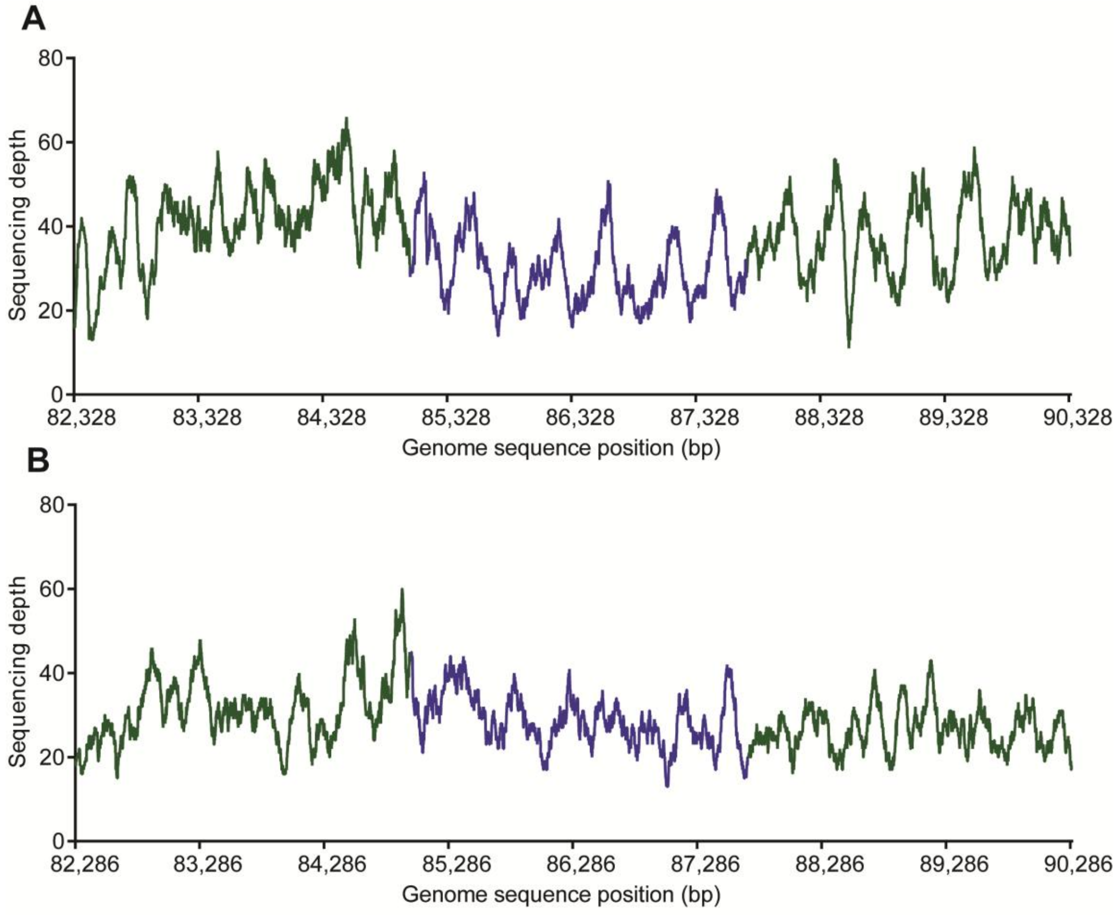

A
80
60
40
20
0
Sequencing depth
82,328
83,328
84,328
85,328
86,328
87,328
88,328
89,328
90,328
Genome sequence position (bp)
B
80
60
40
20
0
Sequencing depth
82,286
83,286
84,286
85,286
86,286
87,286
88,286
89,286
90,286
Genome sequence position (bp)

Figure S2

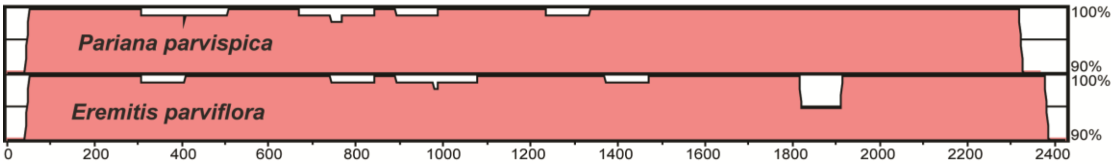
Pariana parvispica
Eremitis parviflora
100%
90%
100%
90%
0
200
400
600
800
1000
1200
1400
1600
1800
2000
2200
2400

Figure S3

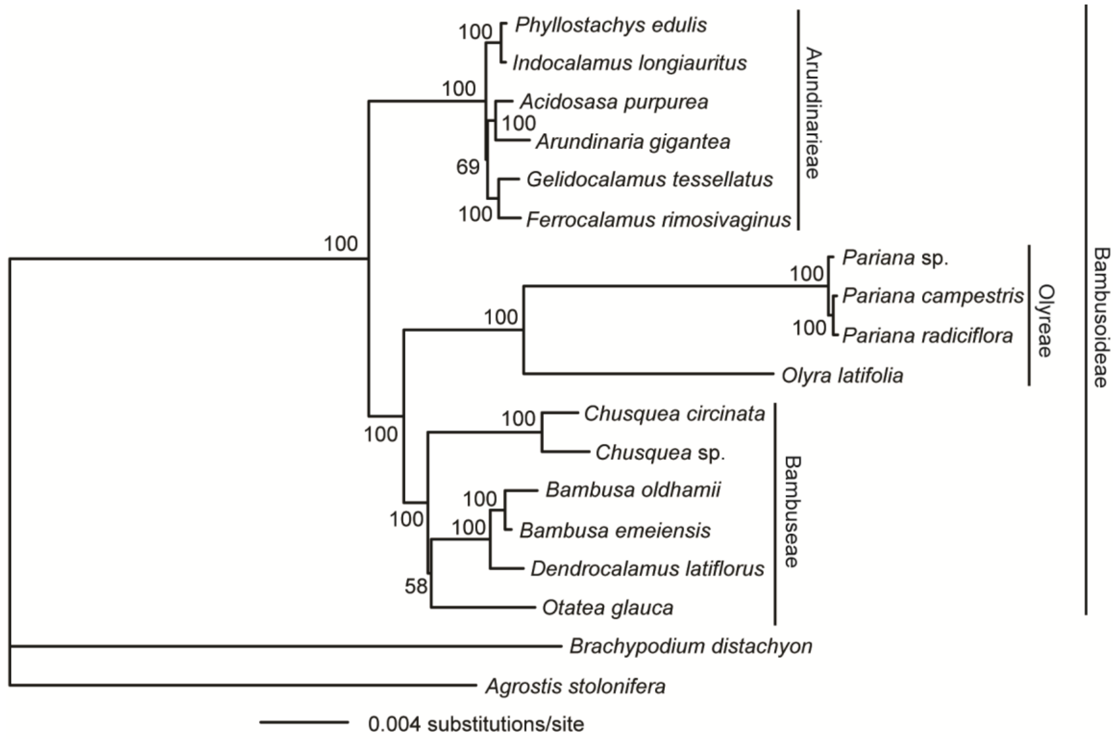
Phyllostachys edulis
Indocalamus longiauritus
Acidosasa purpurea
Arundinaria gigantea
Gelidocalamus tessellatus
Ferrocalamus rimosivaginus
Arundinarieae
Pariana sp.
Pariana campestris
Pariana radiciflora
Olyra latifolia
Olyreae
Chusquea circinata
Chusquea sp.
Bambusa oldhamii
Bambusa emeiensis
Dendrocalamus latiflorus
Otatea glauca
Bambuseae
Bambusoideae
Brachypodium distachyon
Agrostis stolonifera
100
100
100
69
100
100
100
100
100
100
100
100
100
100
100
58
0.004 substitutions/site
